# The Impact of Virtual Reality Distraction on Pain and Anxiety during Dental Treatment in 4-6 Year-Old Children: a Randomized Controlled Clinical Trial

**DOI:** 10.5681/joddd.2012.025

**Published:** 2012-11-12

**Authors:** Naser Asl Aminabadi, Leila Erfanparast, Azin Sohrabi, Sina Ghertasi Oskouei, Armaghan Naghili

**Affiliations:** ^1^Professor, Department of Pediatric Dentistry, Faculty of Dentistry, Tabriz University of Medical Sciences, Tabriz, Iran; ^2^Assistant Professor, Department of Pediatric Dentistry, Faculty of Dentistry, Tabriz University of Medical Sciences, Tabriz, Iran; ^3^Research Assistant, Faculty of Dentistry, Tabriz University of Medical Sciences, Tabriz, Iran; ^4^Dentist, Private Practice, Tabriz, Iran

**Keywords:** Anxiety, dental treatment, distraction, pain, virtual reality

## Abstract

**Background and aims:**

Dental practitioners have numerous methods to control anxiety and pain in children, and distracting the child appears to be the most common technique used for behavior management during dental procedures. The aim of the present study was to evaluate the influence of using virtual reality eyeglasses on severity of pain and anxiety during dental procedures in pediatric patients.

**Materials and methods:**

This study included 120 healthy children aged 4-6 years. Children with no previous anxiety disorder were randomly divided into two groups, each consisting of 60 children. The study consisted of 3 consecutive treatment sessions. During the first visit fluoride therapy was carried out in both groups. In the next sessions, the groups received restorative treatment with and without virtual reality eyeglasses in a randomized single-blind-controlled crossover fashion. Then at the end of each session the subjects’ pain severity was assessed using Wong Baker FACES Pain Rating Scale and state anxiety was measured by Faces version of the Modified Child Dental Anxiety Scale [MCDAS (f)].

**Results:**

There was a significant decrease in pain perception (P < 0.001) and state anxiety scores (P < 0.001) with the use of virtual reality eyeglasses during dental treatment.

**Conclusion:**

Results of this study showed that virtual reality eyeglasses can successfully decrease pain perception and state anxiety during dental treatment. Trial registration number: 201103126036N1.

## Introduction

pain and anxiety are unpleasant feelings and emotional experiences, which are associated with real or possible traumas to tissues.^1^ Management strategies have been proposed to reduce distress during dental treatment in children and are mainly divided into two broad categories. The first module consists of behavioral techniques including the tell-show-do technique, distraction, inspiration, modeling and hypnotism. The second category consists of pharmacologic techniques.^2,3^

Distraction appears to be safe and inexpensive and gives rise to an effective relaxed experience in short painful dental procedure.^2^ Previous studies have shown that distraction is the most common technique used to reduce pain in short invasive medical procedures.^4,5^

The application of distraction is based on the assumption that pain perception has a large psychological component in that the amount of attention directed to the noxious stimuli modulates the perceived pain. Although the precise mechanism of distraction is not yet well understood, cognitive-affective attention models may explain this phenomenon.^6^ McCaul and Mallet^7^ developed the existing theory by placing emphasis on the fact that the capacity of humans to pay attention is limited. They point out that an individual should concentrate on the painful stimuli in order to perceive pain; therefore, perception of pain decreases when a person’s attention is distracted away from the stimulus.^7^

Based on the results of a number of studies the ideal process of distraction requires the capture of the child’s various senses such as vision, hearing and touch and actively engaging the child’s emotions.Therefore the ideal distracter would require an optimal amount of attention involving multiple sensory modalities (visual, auditory, and kinesthetic), active emotional involvement and participation of the patient to compete with the signals from the noxious stimuli.^8,9^

Previous techniques to distract a child include watching television, listening to music, counting the furniture in the room and non-medical dialogs, which serve to distract the child’s attention from anxiety-provoking stimuli.^8^ Comparing three distraction techniques for reducing stress in patients, Seyrek et al.^10^ found that video techniques were more effective than an audio program. Results further suggested that successful distraction was accompanied by an increase in physiological arousal, possibly indicating the degree of psychological absorption or engagement in the video.

In recent years, there has been an increase in behavioral research in virtual reality (VR) and virtual world. VR refers to a human–computer interface that enables the user to interact dynamically with the computer-generated environment. In contrast to the less complex audiovisual (A/V) distraction, VR uses sophisticated systems such as head-mounted, wide field-of-view; three-dimensional displays (HMDs) and motion sensing systems that measure the user’s head and hand positions. This application may be superior to traditional distraction because it offers more immersive images due to the occlusive headsets that project the images right in front of the eyes of the user and, depending on the model used, block out real-world (visual, auditory, or both) stimuli. VR even combines the audio, visual, and kinesthetic sensory modalities. Depending on how immersive the presented stimuli are, the person’s attention will be more or less “drained” from the real world, leaving less attention available to real-world processes, including painful stimuli. Immersion is particularly increased during VR because the use of HMDs prevents patients from seeing what is happening in the real world and directs the focus on what is going on in the virtual world. Therefore, the child’s attention is focused on what happen in the virtual world rather than on the surrounding environment.^8^ Sullivan et al.^11^ demonstrated that using virtual reality during dental treatment had no significant effect on the behavior or anxiety but significantly reduced the pulse.

Literature review reveals sparse investigations regarding the use of VR technique in children. To date, there have been no studies evaluating the effect of VR distraction on the pain perception and state anxiety in children considering primary childhood anxiety disorders as an important confounding variable. In the present study, an attempt was made to eliminate the effects of childhood anxiety disorders during the use of VR distraction during routine dental treatment.

The objective of this study was to answer the question, as to whether VR distraction is effective in reducing pain and anxiety as adjunct to traditional behavior management strategies in the dental setting and procedures.

## Materials and Methods

This single-blind crossover clinical trial study was carried out in the Department of Pediatric Dentistry at Tabriz University of Medical Sciences. Ethical approval for the study was obtained from the Research Ethics Committee of Tabriz University of medical Sciences (Ref: 89.50).

The participants consisted of 120 children between 4-6 years of age who attended Department of Pediatric Dentistry of Tabriz University of Medical Sciences for routine dental care from November 2011 until March 2012. Children who did not have anxiety disorders at the first attendance according to SCARED questionnaire were included in the study. Other inclusion criteria were the first attendance and presence of at least two carious mandibular primary molars requiring restorative treatment.

From the 250 children matching the inclusion criteria, 120 subjects were randomized into two groups. A random allocation list was generated using a randomization software (RandList version 1.2; DatIng GmbH, Tübingen, Deutschland; seed number: 1,901,365,632) to allocate subjects to any of the groups, one by one according to their order of admission.

The treatment consisted of three consecutive sessions. All dental procedures were carried out by one pediatric dentist. The VR device (i-glasses 920HR Ilixco, Inc. Menlo Park, CA, USA) used during the dental procedures blocked the visual field of the child completely and had headphones to deliver the sound. The device was connected to a player capable of playing MP4 audio-visual files. A single episode of the cartoon series “Tom and Jerry” was played for all subjects throughout the study.

In the first session, all the children in both groups received fluoride therapy without any intervention. In the second session VR device was introduced to the subjects in group 1 using tell-show-do technique before treatment. Once VR device was adopted on the child’s eyes, playing the cartoon was started. Then, topical anesthetic agent was placed by a piece of cotton roll on the injection site,^12^ and inferior alveolar block injection was administered, followed by a primary mandibular molar restoration. Subjects in group 2 received similar procedures without the use of VR distraction. In the third appointment which took place 1 to 2 weeks after the second session, primary mandibular molar restoration with inferior alveolar nerve block injection was performed with and without VR distraction in groups 2 and 1, respectively. Each therapeutic session lasted about half an hour.

### The Instruments

**Screen for Child Anxiety Related Disorders (SCARED) Questionnaire:** The parent version of this questionnaire has been designed to evaluate symptoms as a result of separation anxiety, overall anxiety, phobic disorders, compulsive disorders, fear of trauma, social phobia, specific phobia and fear of school in children under 8 years of age. The questionnaire was used to evaluate the presence of childhood background anxiety disorders in the subjects. In this questionnaire, scores above 25 indicate the presence of childhood anxiety disorders and were excluded from the study.^13^ The Persian format of the questionnaire had been used in previous studies.^14^

**Faces version of the Modified Child Dental Anxiety Scale [MCDAS(f)] Questionnaire:** This questionnaire is used for evaluating state anxiety in wide age range in children during dental procedures.^15^

**Figure 1:**
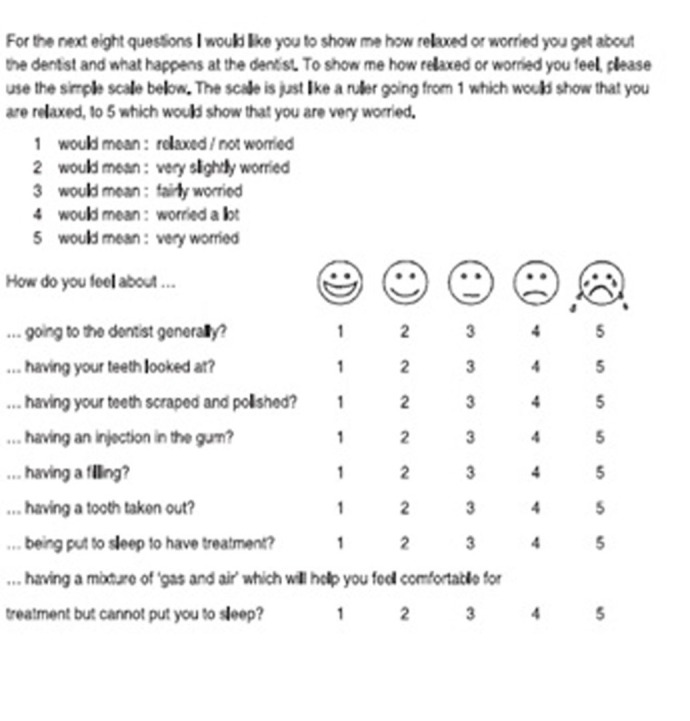
Faces version of the Modified Child Dental Anxiety Scale [MCDAS(f)] Questionnaire.

This index is self-reported and consists of 8 questions with 5 pictorial answers for each question. Scores on the MCDAS(f) scale may range from 8 to 40, with scores below 19 indicating absence of state anxiety, scores higher than 19 indicating the presence of state anxiety and scores higher than 31, indicating severe phobic disorder (Figure 1).^16^ The Persian format of this questionnaire had also been used in previous studies.^14^

**Figure 2:**
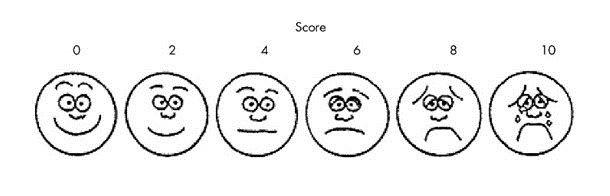
Wong Baker FACES Pain Rating Scale.

**Wong Baker FACES Pain Rating Scale:** This technique was used to assess pain perceived during dental procedures. It consists of a number of faces ranging from happy to crying. The children were asked to indicate the level of pain they perceived on this pictorial index (Figure 2).^17^

Once the treatment is completed, the eyeglasses were removed.^11^ Wong Baker FACES Pain Rating Scale, which is a self-reported scale was explained and shown to the subjects immediately after treatment and they were asked to show the face which best depicted the pain level they experienced during treatment. Faces version of the Modified Children Dental Anxiety Scale [MCDAS (f)] was used to evaluate state anxiety of the subjects in a manner similar to the evaluation of pain.^16^

### Data Analysis

Data were analyzed using SPSS version 15.0 software (SPSS Inc.). Chi-square or Fisher’s Exact Test was used to assess gender difference between the two groups. Mann-Whitney U test was used to compare age and anxiety disorders (SCARED) scores differences between the two groups. Paired samples and independent sample t-tests were used to compare the level of pain and state anxiety. The Kappa statistic was calculated for inter- and intra-rater reliability assessments. The statistical significance was set to 0.05.

## Results

Three of the 120 subjects failed to attend the second and third treatment sessions, leaving a total of 117 subjects in the present study. The overall mean age of patients was 5.4 years (range, 4-6). The mean ages of the subjects in groups 1 and 2 were 5.18 ± 0.67 and 5.65 ± 0.71 years, respectively. No significant difference was seen in the means of ages between the two groups (P = 0.81).

The subjects comprised 33 boys and 25 girls in group 1 and 30 boys and 29 girls in group 2, with no statistically significant differences between the two groups regarding gender (Fisher’s Exact test, P = 0.75).

The mean SCARED score was 16.74 ± 1.52 in groups 1 and 16.65 ± 2.03 in group 2 Childhood Anxiety-Related Disorders scores did not differ significantly between the two groups (Mann-Whitney U test, P = 0.81).

**Figure 3:**
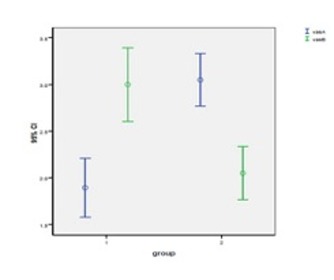
The error bars of pain severity as determined by faces scale in the two groups under study. Group 1: VR device used in the second appointment; Group 2: VR device used in the third appointment.

In group 1, the mean faces scale pain scores in the first (with VR distraction) and second (without VR distraction) treatment sessions were 1.89 ± 0.65 and 3.00 ± 0.81, respectively. These values represent a statistically significant increase in pain score. In group 2, the mean of faces scale pain score was 3.05 ± 0.60 in the first treatment session (without VR distraction), which decreased to 2.05 ± 0.60 in the second session (with VR distraction). In both groups, a statistically significant difference was detected between the two treatment sessions (P < 0.001; Figure 3).

**Figure 4:**
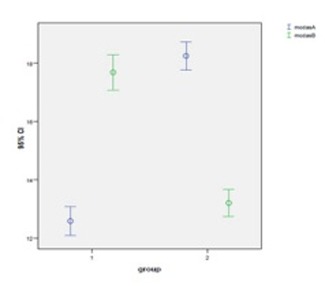
The error bars of state anxiety severity as determined by MCDAS(f) in the two groups under study. Group 1: VR device used in the second appointment; Group 2: VR device used in the third appointment.

Similarly, in group 1 the Mean MCDAS (f) anxiety scores in the first (with VR distraction) and second (without VR distraction) treatment sessions were 12.58 ± 1.01 and 17.68 ± 1.25, respectively. These values represent a statistically significant increase in anxiety score. In group 2, the Mean MCDAS (f) anxiety scores was 18.25 ± 1.02 in the first treatment session (without VR distraction), which decreased to 13.20 ± 1.00 in the second treatment session (with VR distraction). In both groups, a statistically significant difference was detected between the two treatment sessions (P < 0.001; Figure 4).

## Discussion

This study tested the effects of distraction using virtual-reality technology on pain perception and anxiety level in children who were primarily screened in order to childhood anxiety related disorders as an important confounding factor in the dental setting. Dental anxiety which is an example of state anxiety might be influenced by trait anxiety. Trait anxiety refers to a general level of stress that is characteristic of an individual, that is, a trait related to personality; and has a constant level during the life span.^18^ It has been shown that patients who have a high level of trait anxiety usually report higher levels of anxiety and pain during dental procedures,^19,20^ and do not respond well to distraction techniques.^21^ To the best of our knowledge previous studies have not taken into account the influence of the issue and therefore, in our study, SCARED questionnaire was used to exclude the children who had anxiety disorders as a confounding affects of anxious personality on dental anxiety.^18^

Since individuals have different pain thresholds, the present study was designed as a crossover study so that each individual would be compared with themselves in two different situations and, therefore the differences in pain threshold would not result in bias in reporting the results. Moreover, unpleasant pain experience can increase pain perception and anxiety during the next sessions, resulting, in turn, to perceive more pain.^20^ It has also been demonstrated that distraction techniques are less effective in individuals who have a previous bitter pain experience.^9^ Therefore, in the present study subjects were excluded if they had previous invasive painful medical or dental history.

The results of the present study showed that use of VR distraction was effective in decreasing pain perception and state anxiety level in children without anxiety disorders during routine dental treatment. In same line, review of the literature revealed a decrease in the stress levels in the majority of studies using VR distraction.^2,22,23^ Several studies have shown that VR distraction has positive effect on pain, anxiety and behavior during medical procedures such as treatment of traumatic injuries, burn care, dental procedures, chemotherapy, injection or blood sampling, and physiotherapy.^2,22-29^ These benefits may be related to more immersive images due to the occlusive headsets that project the images right in front of the eyes of the user and block out real-world (visual, auditory, or both) stimuli. The child’s attention is focused on what happen in the virtual world rather than on the surrounding environment.^8^ VR even combines the audio, visual, and kinesthetic sensory modalities. Therefore VR, being the most immersive of all and depending on how immersive the presented stimuli are, the person’s attention will be more or less “drained” from the real world, leaving less attention available to process other real-world, including painful stimuli.^11,24^ The application of VR distraction is based on the assumption that pain perception has a large psychological component and that pain attracts a strong attentive response because of the potential threat of damaged tissue associated with the sensation. The redirection (distraction) of this attention manipulates the pain perception, thereby reducing the intensity of pain. Recently it has also been found that VR changes the way people interpret incoming pain signals and actually reduces the amount of pain-related brain activity.^30^ Moreover it can be concluded that VR engages the conscious attention of the patient, resulting in less pain perception by the patients.^24^ By diverting attention from an unpleasant medical setting to a pleasant and absorbing virtual world, while also engaging higher cognitive and emotional centers of the nervous system, VR can markedly diminish a patient’s subjective pain experience.^30^

In addition, researchers have evaluated the neurobiological mechanism of VR technique in the brain by fMRI and have concluded that the effect of VR technique on pain perception is beyond simple distraction. ^31^ VR technique produces a deep illusion of entering a virtual world produced by a computer through coordination of sensory perceptions (vision, hearing and sometimes touch), which is referred to as “presence”. Presence forms a basis for VR technique. In fact, the level of presence in the virtual world reflects the amount of attention that individual directs towards the virtual atmosphere; the more an individual is absorbed in the virtual world, the less he/she is expected to perceive pain.^32^ Moreover using fMRI to monitor brain activity during pain distraction, studies have demonstrated that cortical areas associated with attentional processes and pain modulation are more active during distraction, whereas areas associated with pain perception are less active.^33^

Additional advantages for the use of virtual reality include ease of use, greater control of the therapy, safe in the majority of patients, no need for instructing the patients and the therapeutic personnel.^8^ In addition, frequent application of the technique does not decrease its positive effects.^25^ Therefore, it can easily be used in children and with some size modifications in adults.^19^

In accordance with our result, previous studies have shown the efficacy of VR distraction in children. It has shown that VR technique induced a higher level of presence in children compared to adults.^26^ Das et al.^34^ reported that older children considered VR technique as a very simple game and therefore younger children are absorbing too much than older children, and therefore have lower level of distraction. These results place great emphasis on the suitability of the selected VR program for the age and personality traits of the subjects, which allows a child to exercise a higher degree of control over the unpleasant stimulus and to imagine himself or herself in a familiar environment.^2^

However, some contradictory results have also emerged as a result of methodological shortcomings and the use of inappropriate devices. Dahlquist et al^21^ showed that use of VR technique is more effective in older children than in younger children compared to simple distraction techniques. Since the device used had been designed for adults, the headphones did not fit very well on young children and therefore did not block the sound and visual field of the surrounding environment in young children. In the present study, smaller size of the VR device was used to accommodate in children. In addition, these devices frequently have been designed for standing or sitting positions, while the device used in the present study is applicable in supine position for dental procedures.

There are considerations that should be taken into account in virtual reality applications. Hoffman et al^35^ reported that a number of patients undergo emotional and nervous states during the first minutes of VR device use and need more time to adapt themselves to the device. In addition, more attention should be paid to differences of personality traits which lead to emotional states in the individual. Fanurik et al^8^ divided their child subjects into attender and distractor groups. The attender group consisted of children who focused all their attention on the therapeutic process; therefore, lack of the visual field during VR device use meant lack of control for them, which increased their anxiety level. In contrast, distracters were children who focused their attention on processes of the therapeutic procedure; therefore, they experienced less anxiety.^24^

In summary, the findings of the present study confirm the efficacy of VR distraction in the dental setting. The anxiety-inducing appearance of dental equipment and the child’s focusing on all the details of the procedure is one of the most important reasons for stress associated with dental procedures in children. Therefore, the positive effects of VR distraction on the pain and anxiety in children in the present study are attributed to the complete blockage of children’s visual fields, and as a result to a successful distraction technique. Moreover, these benefits may be related to more immersive images due to combination of the audio, visual, and kinesthetic sensory modalities in VR.

A limitation of the present study was the fact that the therapeutic procedures were carried out in two separate sessions. It is important to note that the baseline anxiety level of children might be different in each session due to factors such as lack of sleep. Therefore, the use of a questionnaire before the procedure in each session to determine the baseline anxiety level in children might assist in eliminating a confounding factor.

In addition, since the success of VR technique in distracting the child depends on the appeal of the program played, it is suggested that the patients themselves assist in choosing their favorite programs in order to achieve better results.

Another limitation of the present study was the limited age range of the subjects. Children aged 4-6 years of age were included because it has been reported that children at this age range exhibit the most negative and aberrant behaviors during dental procedures and are the most difficult to control.^2^ However, since different age groups exhibit different cognitive characteristics and behavioral patterns toward VR technique, it is recommended that different age groups be evaluated in future studies. However, considering the fact that children’s anxiety and pain can have different faces with various levels of alarm such as evolving temperament may conspire to affect, positively or negatively, the extrapolation of the results of the present study to a broader sense and generalization of the findings necessitates further investigation. It is also suggested that the efficacy of VR technique be evaluated in other dental procedures such as extraction and pulp therapy.

## Acknowledgments

This study was supported and funded by Tabriz University of Medical sciences. Trial registration number: 201103126036N1. The authors are thankful to the staff at the Department of Pediatric Dentistry for their assistance and the parents and children for participating in the study.
